# Trends in coronary artery disease mortality among hyperlipidemic patients: Geographic, gender, and racial insights from CDC WONDER data (1999–2020)

**DOI:** 10.1016/j.ijcrp.2025.200416

**Published:** 2025-05-03

**Authors:** Muhammad Abdullah Naveed, Sivaram Neppala, Himaja Dutt Chigurupati, Bazil Azeem, Ahila Ali, Faizan Ahmed, Sabin Zafar, Muhammad Omer Rehan, Rabia Iqbal, Manahil Mubeen, Hassaan Abid, Anum Mubasher, Timir Paul

**Affiliations:** a Department of Cardiology, Dow Medical College, Dow University of Health Sciences, Karachi, Pakistan; b Department of Cardiology, University of Texas Health Sciences Center, San Antonio, TX, USA; c Department of Internal Medicine, Saint Micheals Medical Center, Newark, NJ, USA; d Division of Cardiology, Shaheed Mohtarma Benazir Bhutto Medical College Lyari, Karachi, Pakistan; e Department of Internal Medicine, Ameeruddin Medical College, Lahore General Hospital, Lahore, Pakistan; f Department of Internal Medicine, Indiana University Ball Memorial Hospital, Indiana, USA; g Department of Internal Medicine, Texas Health Resources, Bedford, USA; h Department of Cardiovascular Sciences, Ascension St. Thomas Hospital/University of Tennessee Health Sciences Center, Nashville, TN, USA

**Keywords:** Coronary artery disease, Hyperlipidemia, Mortality, Gender, Race, Geographics

## Abstract

**Background:**

Coronary artery disease (CAD) in hyperlipidemia is a significant cause of mortality among adults in the United States. This study investigates trends in CAD-related mortality in hyperlipidemia among adults aged 25 and older, focusing on geographic, gender, and racial/ethnic disparities from 1999 to 2020.

**Methods:**

A retrospective analysis was conducted using the CDC WONDER database from 1999 to 2020. Age-adjusted mortality rates (AAMRs), annual percent change (APC), and average annual percentage change (AAPC) were calculated per 100,000 persons, stratified by year, sex, race/ethnicity, and geographical region.

**Results:**

Between 1999 and 2020, CAD in hyperlipidemia led to 407,667 deaths among US adults aged 25+. The AAMR for CAD in hyperlipidemia rose from 4.1 in 1999 to 12.1 in 2020, with an AAPC of 4.44 (95 % CI: 3.69 to 5.48, p < 0.000001). Men had higher AAMRs (12.4) than women (5.6), with significant increases for both sexes over time. Racial/ethnic disparities showed the highest AAMRs in Whites (8.9), followed by American Indians/Alaska Natives (8.6). Geographically, AAMRs varied from 3.8 in Alabama to 16.0 in Vermont, with the Midwest showing the highest rates (9.7). Nonmetropolitan areas exhibited higher AAMRs (9.6) than metropolitan areas (8.3), with a more significant increase in nonmetropolitan areas (AAPC: 5.82, p < 0.000001).

**Conclusion:**

This study highlights significant disparities in CAD in hyperlipidemia-related mortality among US adults by race, gender, and geography. The overall increase in AAMRs from 1999 to 2020 underscores the need for targeted public health interventions to address these growing inequities.

## Highlights

• The AAMR rose from 4.1 in 1999 to 12.1 in 2020, reflecting an Average Annual Percentage Change (AAPC) of 4.44. This increase was particularly pronounced from 1999 to 2006, with an annual percentage change (APC) of 10.02.
• Gender disparities in AAMR were notably apparent, with adult men consistently exhibiting higher AAMRs than adult women (overall AAMR for men: 12.4 vs. 5.6 for women).
• All racial and ethnic groups experienced varying increases in AAMRs from 1999 to 2020, with the Black population showing the most significant surge.
• The disparities in age-adjusted mortality rates (AAMR) across states were striking, ranging from 3.8 in Alabama to 16.0 in Vermont. The highest mortality rates were observed in the Midwestern region, followed by the Western region.
• From 1999 to 2020, AAMRs significantly increased in metropolitan and nonmetropolitan areas, with a more pronounced increase observed in nonmetropolitan areas.

## 1 Introduction

Hyperlipidemia, characterized by elevated blood lipid levels, represents a significant risk factor for cardiovascular morbidity and mortality by contributing to the accumulation of plaques within the arteries [[1], [2], [3]]. Consequently, there has been a constant emphasis on preventing and managing hyperlipidemia as a crucial risk factor, highlighting its prognostic significance in reducing the burden of CAD [4].

Previous angiography trials have demonstrated that reducing serum cholesterol levels can slow the progression of atherosclerosis, subsequently lowering the risk of CAD [[5], [6], [7], [8], [9]]. Several proposed mechanisms explain how lowering lipid levels can reduce coronary events. These include stabilizing plaques by reducing cholesterol, improving endothelial function, and decreasing platelet thrombus formation due to lower cholesterol levels [10,11]. Studies have shown that individuals with hyperlipidemia exhibit elevated platelet activity, leading to the formation of mural thrombi at the site of platelet rupture [12,13]. Numerous studies have revealed that cardiovascular health is a complex interplay of physiological and behavioral risk factors, 90 % of which are adjustable. These include hyperlipidemia, smoking, stress, diabetes, hypertension, alcohol consumption, and a sedentary lifestyle [14]. The prevalence of poor cardiovascular health has been on the rise nationwide between 2003 and 2011, preceding the recent surge in cardiovascular mortality [15]. Notably, there are significant regional disparities in cardiovascular health, with higher rates of suboptimal cardiovascular health observed in Southern states [16,17].

In addition, a study by Pekkanen, J et al. demonstrated that serum cholesterol levels can predict cardiovascular and coronary heart disease mortality in men without preexisting cardiovascular disease [18]. Moreover, existing literature has highlighted profound racial and ethnic disparities in CAD-related mortality, which are associated with a complex interplay of factors such as socioeconomic status, healthcare access, cultural differences, and the prevalence of comorbid conditions, including hyperlipidemia, diabetes, and hypertension [19].

This study, which included patients from 1999 to 2020, provides valuable insights into the long-term trends and evolving nature of CAD-related mortality in individuals with hyperlipidemia. By comparing these trends with existing literature, the research underscores the urgency for targeted public health strategies and policy interventions that address the specific needs of diverse populations. Reducing these disparities will necessitate a multifaceted approach involving improving healthcare access, education, socioeconomic conditions, and culturally sensitive healthcare practices.

## 2 Methods

### 2.1 Database and cohorts’ definition

This study used the Centers for Disease Control and Prevention's Wide-Ranging OnLine Data for Epidemiologic Research (CDC WONDER) database encompassing data from all 50 states and the District of Columbia. We included adults with hyperlipidemia diagnosis who were aged 25 years or older at the time of their death between 1999 and 2020. We examined death records from the Multiple Causes of Death Public Use registry to identify CAD-related mortality in these patients. CAD-related mortality was either the primary cause of death or a contributing factor. We utilized the diagnostic codes "E78″ and “I25” from the 10th version of the International Classification of Diseases and Related Health Problems (ICD-10). Due to the utilization of deidentified public use data provided by the government, the study did not require approval from a local institutional review board. The study adhered to the STROBE standards for reporting observational research.

### 2.2 Data abstraction

The CDC WONDER database comprised multiple variables collected from death certificates, including demographics such as gender, age, race, ethnicity, year of death, and location. It also includes geographic segmentation, classified per the United States Census Bureau into the Northeast, Midwest, South, and West, state-specific information, and differentiation between urban and rural areas. Death locations include hospitals, private residences, hospices, nursing homes, and long-term care facilities. The population was categorized based on the National Center for Health Statistics Urban-Rural Classification Scheme, dividing it into urban areas with large metropolitan regions (1 million or more population) and medium/small metropolitan areas (populations ranging from 50,000 to 999,999). Rural regions were classified based on the 2013 U S. Census, encompassing areas with fewer than 50,000 inhabitants and additional counties.

### 2.3 Statistical analysis

The CAD-related mortality rates were calculated per 100,000 individuals and categorized by year, gender, race/ethnicity, state, and urban/rural status. The raw mortality rates were determined by dividing the total number of CAD-related deaths in patients with hyperlipidemia by the corresponding U.S. population each year. Age-adjusted mortality rates (AAMRs) were standardized to the 2000 U S. population. The annual percent change (APC) and its associated 95 % confidence interval (CI) in AAMR were computed using the Join-Point Regression Program (Version 5.0.2, National Cancer Institute) to identify annual changes in CAD-related mortality at the national level. This analysis utilized log-linear regression models, classifying the APCs as either increasing or decreasing based on whether the slope reflecting changes in mortality significantly differed from zero, assessed via two-tailed t-testing at a significance level of P < 0.05.

## 3 Results

Between 1999 and 2020, a staggering total of 1,462,279 adults with hyperlipidemia aged 25 years and above in the United States had CAD-related mortality (Supplementary Table 1). These deaths mainly occurred (40.1 %) in medical facilities, followed by 37.3 % at the decedents' homes, 16.4 % in nursing homes/long-term care facilities, 2.1 % in hospice facilities, and 4.0 % at other locations (Supplementary Table 2)**.**

### 3.1 Age-adjusted mortality rate (AAMR)

There was a significant increase in the AAMR for coronary artery disease (CAD) in individuals with hyperlipidemia, rising from 4.1 in 1999 to 12.1 in 2020. This represents an Average Annual Percentage Change (AAPC) of 4.44 (95 % CI: 3.69–5.48). Notably, the AAMR experienced a substantial increase from 1999 to 2006 (APC: 10.02; 95 % CI: 6.71–18.61), followed by a more moderate increase from 2006 to 2020 (APC: 1.76; 95 % CI: 0.92–2.51) (Supplementary Table 3)**.**

### 3.2 Gender analysis

**Fig. 1:**
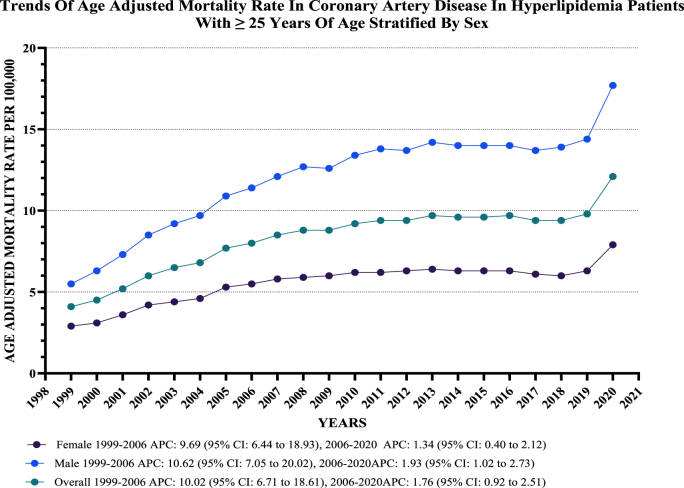
**Age-adjusted mortality Rates per 100,000 Related to CAD in Hyperlipidemia Patients, Stratified by gender in the United States, from 1999 to 2020**.

There was a significant gender disparity in AAMRs, with adult men consistently showing higher AAMRs than adult women (overall AAMR for men: 12.4 vs. 5.6 for women). Both men and women experienced an increase in AAMRs from 1999 to 2020, with the increase being more pronounced in men (Men: Annual Percentage Change (APC): 4.75, 95 % CI: 3.96–5.96; Women: APC: 4.05, 95 % CI: 3.27–5.04). In particular, the AAMR for adult men exhibited a substantial rise from 5.5 in 1999 to 11.4 in 2006 (APC: 10.62, 95 % CI: 7.05–20.02), followed by a moderate increase to 17.7 by 2020 (APC: 1.93, 95 % CI: 1.02–2.73). Similarly, the AAMR for adult women increased from 2.9 in 1999 to 5.5 in 2006 (APC: 9.69, 95 % CI: 6.44–18.93), followed by a modest increase to 7.9 by 2020 (APC: 1.34, 95 % CI: 0.40–2.12) (Fig. 1, Supplementary table: 4).

### 3.3 Race and ethnicity analysis

There were significant disparities in mortality rates among different racial and ethnic groups. Non-Hispanic Whites exhibited the highest AAMR at 8.9, closely followed by American Indians or Alaska Natives at 8.6. Comparatively, Black or African Americans had an AAMR of 7.3, Hispanic or Latinos had a rate of 6.5, and Asian or Pacific Islander populations had an AAMR of 5.9.

**Fig. 2:**
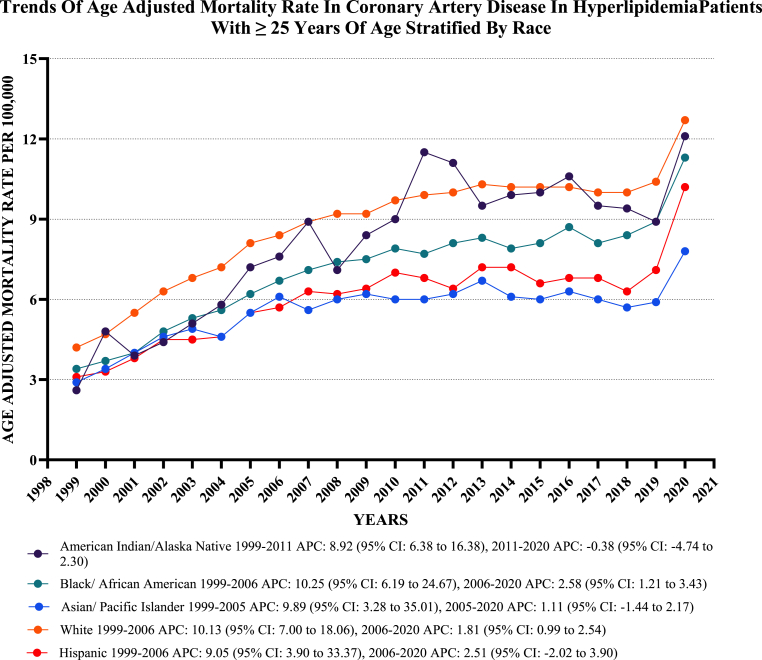
**Age-adjusted mortality Rates per 100,000 Related to CAD in Hyperlipidemia Patients, Stratified by Race in the United States, from 1999 to 2020**.

All racial and ethnic groups had variable increases in AAMRs from 1999 to 2020, with the most noticeable increase observed in the Black population [Black: AAPC: 5.07, 95 % CI: 4.06 to 6.34; American Indian: AAPC: 4.83, CI: 3.60 to 6.93; Hispanic: AAPC: 4.65, CI: 3.22 to 6.76; Non-Hispanic White: AAPC: 4.51, CI: 3.79 to 5.54; American: AAPC: 4.83, CI: 3.61 to 6.93] (Fig. 2, Supplementary Table 5).

### 3.4 Geographic analysis

**Fig. 3:**
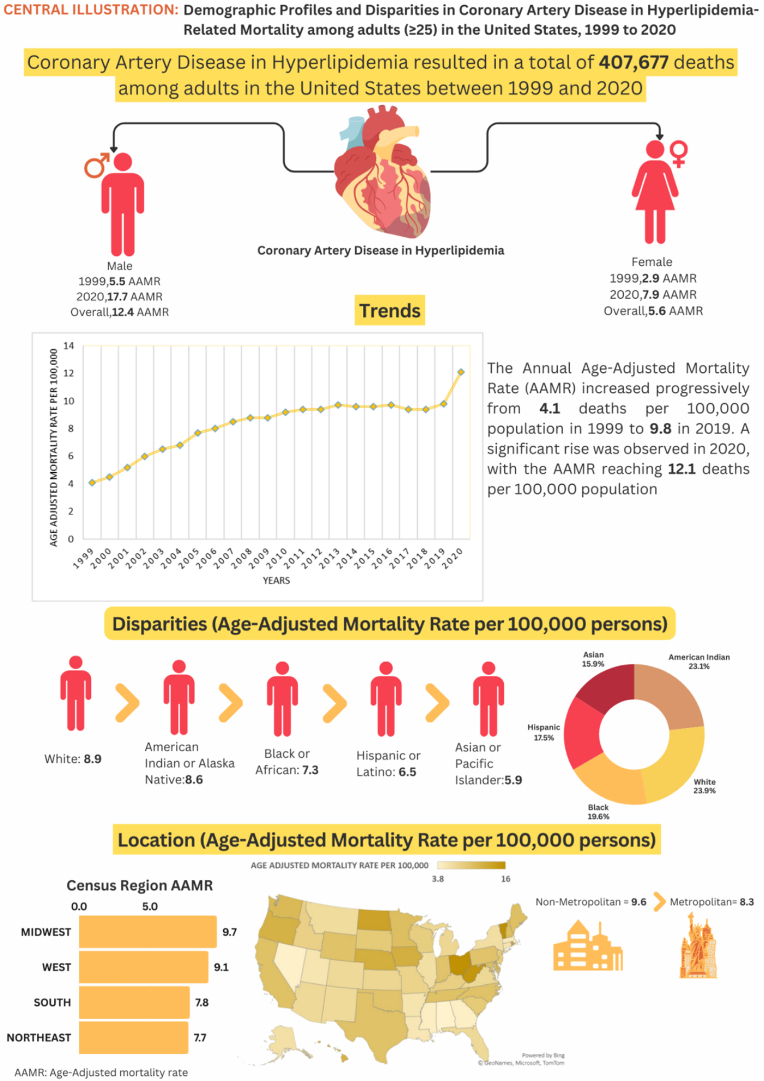
**Central illustration: Trends in demographics and disparities in CAD among adults patients of hyperlipidemia in the United States: 1999 to 2020**.

Variations in AAMRs were noted among states, with rates ranging from as low as 3.8 (95 % CI: 3.7–4.0) in Alabama to 16.0 (95 % CI: 15.3–16.8) in Vermont. States in the top 90th percentile, including Iowa, Nebraska, North Dakota, Ohio, Oregon, Rhode Island, Vermont, and West Virginia, exhibited AAMRs approximately twice as high as states in the lower 10th percentile, such as Alabama, Alaska, Connecticut, Georgia, Louisiana, Massachusetts, Mississippi, Nevada, and Utah (Fig. 3, Supplementary Table 6).

**Fig. 4:**
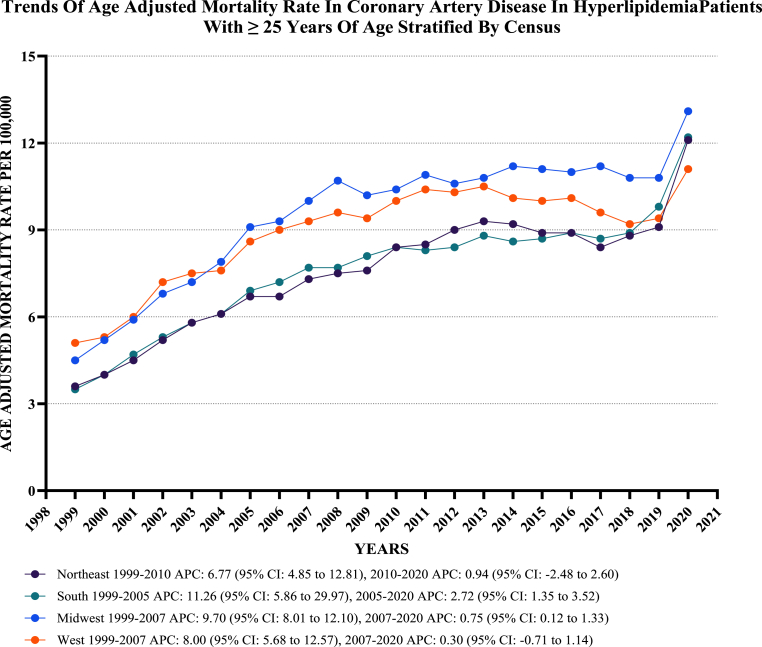
**Age-Adjusted Mortality Rates per 100,000 Related to CAD in Hyperlipidemia Patients, Stratified by regions in the United States, from 1999 to 2020**.

On average, the highest mortality rates were observed in the Midwest (AAMR: 9.7; 95 % CI: 9.6 to 9.7), followed by the West (AAMR: 9.1; 95 % CI: 9.1 to 9.2), the South (AAMR: 7.8; 95 % CI: 7.8 to 7.9), and the Northeast (AAMR: 7.7; 95 % CI: 7.6 to 7.7) (Fig. 4, Supplementary Table 7).

**Fig. 5:**
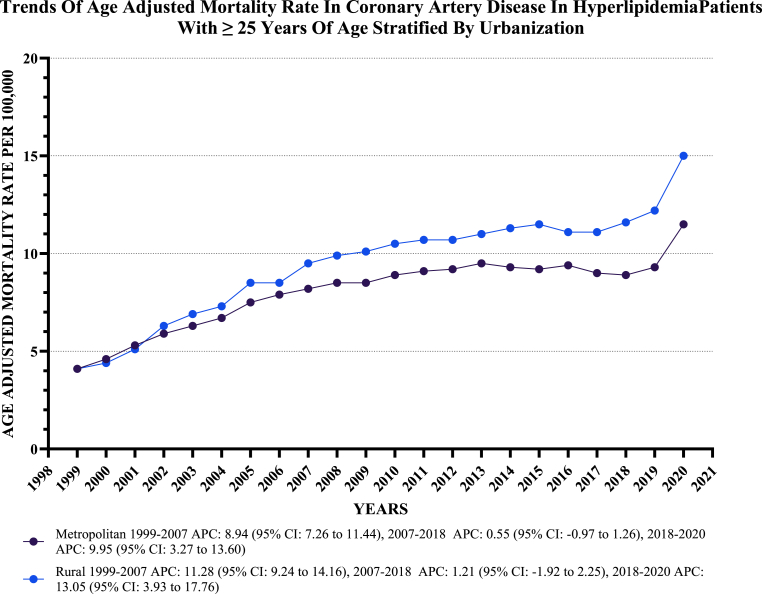
**Age-Adjusted Mortality Rates per 100,000 Related to CAD In Hyperlipidemia Patients, Stratified by urban vs rural regions in the United States, from 1999 to 2020**.

Nonmetropolitan areas showed slightly higher AAMRs than metropolitan areas, with overall AAMRs of 9.6 (95 % CI: 9.6 to 9.7) and 8.3 (95 % CI: 8.2 to 8.3), respectively. Both metropolitan and nonmetropolitan areas experienced a significant increase in AAMRs from 1999 to 2020, with a more significant increase observed in nonmetropolitan areas [Metropolitan: AAPC: 4.17, (CI: 3.46 to 5.14) (p-value <0.000001); Nonmetropolitan: AAPC: 5.82, (CI: 5.17 to 6.60) (P value < 0.000001)] (Fig. 5, Supplementary Table 8).

#### 3.4.1 Comparison of CAD vs CAD with hyperlipidemia

**Fig. 6:**
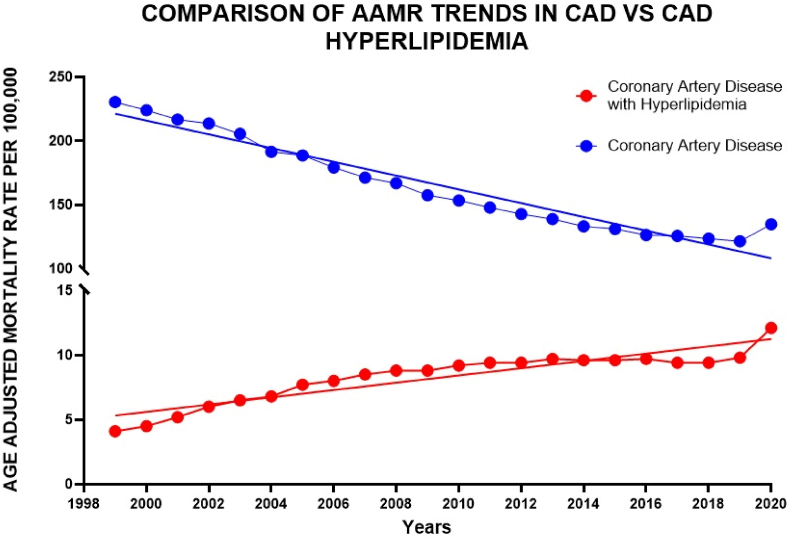
**Comparison of Age-Adjusted Mortality Rates per 100,000 in CAD patients vs. CAD patients with hyperlipidemia**.

The AAMR for CAD in individuals with hyperlipidemia soared from 4.1 in 1999 to 12.1 in 2020, indicating an AAPC of 4.44 (95 % CI: 3.69–5.48). There was a notable surge in AAMR from 1999 to 2006 (APC: 10.02; 95 % CI: 6.71–18.61), followed by a more modest increase from 2006 to 2020 (APC: 1.76; 95 % CI: 0.92–2.51), all p < 0.01. In contrast, the AAMR among the general CAD population significantly declined from 230.4 in 1999 to 134.4 in 2020, with an AAPC of −2.69 (95 % CI: −2.95 to −2.53). The AAMR saw a considerable drop from 1999 to 2012 (APC: −3.80; 95 % CI: −5.06 to −2.72), followed by a moderate decline from 2012 to 2018 (APC: −2.65; 95 % CI: −3.69 to −1.33). However, it sharply increased from 2018 to 2020 (APC: 4.64; 95 % CI: 1.03 to 6.81), all p < 0.01 (Fig. 6).

## 4 Discussion

Our study provides empirical evidence regarding the impact of coronary artery disease (CAD) in individuals with Hyperlipidemia (HLD) across various demographic groups in the United States from 1999 to 2020. The findings underscore the alarming statistic of 1,462,279 deaths among individuals aged 25 and above with CAD and hyperlipidemia during this period, with a significant proportion occurring in medical facilities at 40.1 %. Notably, the overall age-adjusted mortality rate (AAMR) for CAD in hyperlipidemia surged from 4.1 in 1999 to 12.1 in 2020. Men experienced a substantial increase in AAMR compared to women, with rates of 12.4 % and 5.6 %, respectively. Non-Hispanic Whites had the highest AAMR at 8.9 %, closely followed by American Indians or Alaska Natives at 8.6 %. Moreover, the black population displayed the highest rise in AAMR, with an annual percentage change of 5.07 %, and the Midwest and the West had the highest observed mortality rates at 9.7 % and 9.1 %, respectively.

The existing body of literature widely recognizes that hyperlipidemia significantly increases the risk of developing coronary artery disease (CAD) [20,21]. Our study unveiled a notable rise in mortality rates related to CAD among hyperlipidemic patients aged 25 and above between 1999 and 2020. This increase is particularly concerning compared to previous study findings, which indicated a substantial decrease in cardiovascular disease (CVD) mortality rates until 2020, followed by a sustained high level in 2022 [22]. The recent upsurge in mortality, despite advancements in diagnostic and screening technologies, presents a problematic situation. Moreover, additional studies have underscored a consistent increase in cardiovascular disease (CVD) mortality during and after the initial phase of the COVID-19 pandemic, with a majority of deaths attributed to heart diseases in the United States [23]. The initiation of atherosclerotic plaque buildup in adolescence highlights the necessity of early CAD diagnosis among young adults. This problem is further compounded by the escalating global prevalence of childhood obesity [24]. Furthermore, individuals with established risk factors, such as familial hyperlipidemia, face an elevated risk of developing CAD [25].

Our findings also indicate gender disparities in CAD-related mortality, showing an increasing trend in mortality for men compared to women. Men are depicted as having nearly double the risk of myocardial infarction compared to women, and this disparity persists throughout life, albeit diminishing with age [26,27]. The age of onset of cardiovascular events also varies between genders, with the first occurring approximately ten years later in women than in men [26,28]. However, various studies have demonstrated that women present with a higher burden of disease and comorbid conditions and experience worse outcomes. Additionally, women are often underdiagnosed and receive less evidence-based medical care, leading to higher death rates for females following acute myocardial infarction [29]. Research indicates that up to 69 % of the mortality disparity between men and women could be addressed by ensuring that both sexes receive optimal quality of care [30]. Furthermore, biological differences, such as higher lipoprotein A levels in women, significantly influence the prevalence of CAD, as studies have documented that these levels are approximately 5–10 % higher in women than in men, with the level rising during pregnancy and at menopause [31,32].

Ethnic and racial disparities in cardiovascular disease mortality are widespread in the United States, as indicated by various studies [28,33]. Our analysis reveals elevated mortality trends among the white population while contrasting research shows that Black adults experience higher mortality rates due to cardiovascular disease [34,38,39]. In addition, socioeconomic barriers, poverty, and chronic stress are more prevalent among the black population compared to the white population [34,40]. Furthermore, African Americans bear a substantial burden of cardiovascular disease risk factors such as obesity, hypertension, and diabetes [35]. However, they are less likely to have elevated hypertriglyceridemia and are often underdiagnosed with metabolic syndrome [36]. Consistent findings are reported by the National Health and Nutrition Examination Survey (NHANES), which found that compared to whites, blacks have lower triglyceride levels and higher high-density lipoprotein cholesterol (HDL) levels [37].

Disparities in mortality between rural and urban areas in the United States have been extensively documented, with higher mortality rates observed among rural inhabitants compared to urban populations [41,42]. Multiple studies have attributed this disparity to socioeconomic deprivation, limited access to healthcare, and lifestyle factors prevalent in rural areas [[43], [44], [45]].

Geographic disparities in the United States reveal the highest mortality rates in the Midwestern region and the lowest in the Northeastern region. Additionally, states in the top 90th percentile, including Iowa, Nebraska, North Dakota, Ohio, Oregon, Rhode Island, Vermont, and West Virginia, exhibit approximately twice the mortality rate compared to states in the lower 10th percentile. This aligns with other studies on heart failure mortality, which also indicate the highest mortality trends in the Midwest and Southern regions [37,[46], [47], [48]]. Numerous factors contribute to these disparities, such as the increased prevalence of hypertension, diabetes, and obesity in Southern states.

Previous research has consistently documented a significant decrease in mortality associated with coronary artery disease (CAD) over the past few decades. This decline is primarily attributed to advancements in medical therapies, lipid-lowering agents' introduction, and lifestyle choice improvements [49,50]. However, our findings reveal a contrasting trend among individuals with hyperlipidemia, where CAD-related mortality has markedly increased. Additional studies have indicated that, despite overall progress, subpopulations with metabolic risk factors continue to face deteriorating outcomes due to inequities in prevention and treatment [51]. The rising age-adjusted mortality rate (AAMR) among hyperlipidemic patients corroborates existing research that underscores the residual risk of CAD, even with statin therapy [5]. These results underscore the urgent need for more aggressive lipid management and tailored interventions targeting high-risk populations.

Addressing the rising rates of cardiovascular disease mortality requires immediate action to halt the progression. Apparent demographic and geographic variations are evident across the USA, necessitating state-level policies to target awareness and implement screening programs to address the rising mortality trends and existing disparities.

## 5 Limitations

This study has several limitations, primarily stemming from its retrospective design. The use of data from death certificates in the CDC WONDER database introduces the potential for inaccuracies in diagnoses, leading to misclassification bias. Furthermore, the absence of laboratory values for the lipids and clinical data pertaining to general health conditions, comorbidities, and treatment hinders a comprehensive understanding of elevated mortality patterns. Nevertheless, efforts have been made to validate the data to uphold the credibility of our findings.

A significant limitation of our study is that the CDC WONDER database supplies mortality data but lacks information on disease prevalence. Consequently, while we were able to analyze age-adjusted mortality rates related to CAD in patients with hyperlipidemia, we could not evaluate the prevalence of hyperlipidemia or the distribution of various lipid abnormalities among the population. Furthermore, the database does not include data on other key cardiovascular risk factors like diabetes, smoking, hypertension, and obesity, which might have affected the mortality trends observed. This constraint limits our capacity to comprehensively assess the effects of these comorbidities on CAD-related mortality in the hyperlipidemic population.

## 6 Conclusion

There is a rising trend in CAD-related mortality in adult patients with hyperlipidemia, especially. It's important to note that the White population experiences higher mortality rates, contradicting the common belief that African Americans are more affected. Despite facing higher risk factors and enduring structural and environmental racism, African Americans are not experiencing higher mortality rates as expected. The Midwest and the West regions have the highest observed mortality rates, with Alabama found to have higher mortality risks and Vermont showing the lowest mortality risk. This underscores the urgent need for state-level policies to implement widespread awareness and screening programs targeted at counties with elevated mortality rates.

## CRediT authorship contribution statement

**Muhammad Abdullah Naveed:** Writing – original draft, Visualization, Validation, Methodology, Formal analysis, Conceptualization. **Sivaram Neppala:** Writing – review & editing, Writing – original draft, Supervision, Methodology. **Himaja Dutt Chigurupati:** Writing – review & editing, Supervision, Methodology. **Bazil Azeem:** Formal analysis, Data curation. **Ahila Ali:** Writing – original draft, Methodology, Formal analysis. **Faizan Ahmed:** Formal analysis, Data curation. **Sabin Zafar:** Visualization, Formal analysis. **Muhammad Omer Rehan:** Formal analysis, Data curation. **Rabia Iqbal:** Visualization, Validation, Data curation. **Manahil Mubeen:** Visualization, Validation. **Hassaan Abid:** Visualization, Supervision. **Anum Mubasher:** Visualization, Validation. **Timir Paul:** Writing – review & editing, Supervision.

## Declaration of competing interest

None of the authors have any conflict of interest.
