# Supplementary material for: Trends in coronary artery disease mortality among hyperlipidemic patients: Geographic, gender, and racial insights from CDC WONDER data (1999–2020)
Source: Int J Cardiol Cardiovasc Risk Prev. 2025 May 3;25:200416. doi: 10.1016/j.ijcrp.2025.200416 (PMC12138564; doi:10.1016/j.ijcrp.2025.200416)
Supplement: Multimedia component 1 [file mmc1.docx]

**SUPPLEMENTARY TABLES:**

**Supplemental Table 1: Coronary Artery Disease related mortalities in Adults with Hyperlipidemia, Stratified by Sex and Race, in the United States, 1999 to 2020.**

| **Deaths** | | | | | | | | | |
| --- | --- | --- | --- | --- | --- | --- | --- | --- | --- |
| **Year** | **Overall** | **Women** | **Men** | **White** | **Black or African American** | **Asian or Pacific Islander** | **American Indian or Alaska Native** | **Hispanic or Latino** | **Population** |
| **1999** | 7190 | 3058 | 4132 | 6253 | 508 | 119 | 23 | 275 | 180408769 |
| **2000** | 8096 | 3330 | 4766 | 7030 | 558 | 146 | 41 | 305 | 181984640 |
| **2001** | 9479 | 3876 | 5603 | 8240 | 619 | 190 | 34 | 378 | 184305128 |
| **2002** | 11147 | 4549 | 6598 | 9631 | 770 | 228 | 39 | 451 | 186208028 |
| **2003** | 12168 | 4879 | 7289 | 10521 | 846 | 252 | 49 | 477 | 188090429 |
| **2004** | 12969 | 5226 | 7743 | 11231 | 919 | 244 | 49 | 513 | 190205384 |
| **2005** | 15026 | 6149 | 8877 | 12925 | 1035 | 318 | 69 | 644 | 192551384 |
| **2006** | 15849 | 6442 | 9407 | 13565 | 1134 | 363 | 71 | 698 | 195019359 |
| **2007** | 17138 | 7006 | 10132 | 14640 | 1249 | 359 | 87 | 793 | 197403777 |
| **2008** | 18112 | 7287 | 10825 | 15456 | 1337 | 402 | 72 | 823 | 199795090 |
| **2009** | 18511 | 7518 | 10993 | 15690 | 1373 | 432 | 87 | 899 | 202107016 |
| **2010** | 19767 | 7896 | 11871 | 16751 | 1454 | 435 | 98 | 1004 | 203891983 |
| **2011** | 20721 | 8086 | 12635 | 17520 | 1517 | 471 | 127 | 1060 | 206592936 |
| **2012** | 21356 | 8445 | 12911 | 17999 | 1618 | 513 | 131 | 1046 | 208826037 |
| **2013** | 22536 | 8788 | 13748 | 18808 | 1731 | 601 | 116 | 1235 | 211085314 |
| **2014** | 22939 | 8874 | 14065 | 19131 | 1708 | 585 | 132 | 1331 | 213809280 |
| **2015** | 23316 | 9002 | 14314 | 19406 | 1788 | 628 | 141 | 1278 | 216553817 |
| **2016** | 23957 | 9222 | 14735 | 19670 | 1992 | 685 | 145 | 1419 | 218641417 |
| **2017** | 23856 | 9049 | 14807 | 19569 | 1917 | 702 | 143 | 1464 | 221447331 |
| **2018** | 24451 | 9066 | 15385 | 20059 | 2052 | 693 | 146 | 1436 | 223311190 |
| **2019** | 26146 | 9731 | 16415 | 21267 | 2244 | 763 | 141 | 1672 | 224981167 |
| **2020** | 32947 | 12402 | 20545 | 26184 | 2947 | 1062 | 202 | 2484 | 226635013 |
| **Total** | **407,677** | **159,881** | **247,796** | **341,546** | **31,316** | **10,191** | **2,143** | **21,685** | **4,473,854,489** |

**Supplemental Table 2: Coronary Artery Disease Related Mortalities stratified by Place of Death in Adults with Hyperlipidemia in the United States, 1999 to 2020.**

| **Deaths** | | | | |
| --- | --- | --- | --- | --- |
| **Year** | **Medical Facility** | **Nursing Home/Long-term Care Facility** | **Hospices** | **Home** |
| **1999** | 4112 | 646 | - | 2235 |
| **2000** | 4558 | 822 | - | 2505 |
| **2001** | 5102 | 967 | - | 3134 |
| **2002** | 5769 | 1321 | - | 3655 |
| **2003** | 6010 | 1545 | - | 4179 |
| **2004** | 6286 | 1651 | 20 | 4488 |
| **2005** | 6930 | 2106 | 93 | 5320 |
| **2006** | 7141 | 2345 | 120 | 5666 |
| **2007** | 7558 | 2707 | 198 | 6049 |
| **2008** | 7808 | 2882 | 273 | 6311 |
| **2009** | 7733 | 2999 | 261 | 6554 |
| **2010** | 8166 | 3319 | 295 | 7180 |
| **2011** | 8399 | 3574 | 402 | 7525 |
| **2012** | 8299 | 3823 | 443 | 7871 |
| **2013** | 8543 | 4014 | 515 | 8492 |
| **2014** | 8424 | 4202 | 602 | 8814 |
| **2015** | 8343 | 4392 | 618 | 8997 |
| **2016** | 8419 | 4420 | 749 | 9365 |
| **2017** | 8144 | 4420 | 754 | 9455 |
| **2018** | 8196 | 4628 | 892 | 9659 |
| **2019** | 8758 | 4554 | 1070 | 10589 |
| **2020** | 10603 | 5547 | 1195 | 14036 |
| **Total** | **163,301** | **66,884** | **8,500** | **152,079** |

**Supplemental Table 3: Annual percent change (APC) of Coronary Artery Disease Related Age-Adjusted Mortality Rates per 100,000 in Adults with Hyperlipidemia in the United States, 1999 to 2020.**

| **Year Interval** | **APC (95% CI)** | **P-value** |
| --- | --- | --- |
| **Overall** | | |
| 1999-2005 | 11.21 (9.31 to 14.69) | < 0.000001 |
| 2005-2013 | 2.78 (1.94 to 4.03) | < 0.000001 |
| 2013-2018 | -1.25 (-4.54 to 0.01) | 0.05119 |
| 2018-2020 | 13.25 (8.95 to 17.12) | < 0.000001 |
| **Men** | | |
| 1999-2005 | 1.27 (9.38 to 15.97) | 0.0004 |
| 2005-2011 | 3.94 (2.40 to 6.03) | < 0.000001 |
| 2011-2018 | -0.25 (-3.07 to 0.56) | 0.45151 |
| 2018-2020 | 12.31 (7.33 to 15.72) | < 0.000001 |
| **Women** | | |
| 1999-2006 | 9.58 (8.08 to 12.34) | 0.0004 |
| 2006-2013 | 1.86 (0.97 to 3.89) | < 0.000001 |
| 2013-2018 | -1.70 (-5.11 to -0.38) | 0.017996 |
| 2018-2020 | 14.09 (9.38 to 18.18) | < 0.000001 |
| **NH White** | | |
| 1999-2005 | 11.26 (9.49 to 14.47) | < 0.000001 |
| 2005-2013 | 2.91 (2.14 to 4.00) | < 0.000001 |
| 2013-2018 | -1.17 (-4.27 to -0.04) | 0.042791 |
| 2018-2020 | 12.59 (8.61 to 16.02) | < 0.000001 |
| **NH Black or African American** | | |
| 1999-2007 | 10.00 (8.23 to 12.59) | < 0.000001 |
| 2007-2018 | 1.13 (0.07 to 1.80) | 0.040392 |
| 2018-2020 | 14.45 (8.36 to 18.11) | < 0.000001 |
| **NH American Indian or Alaska Native** | | |
| 1999-2011 | 8.92 (6.38 to 16.38) | < 0.000001 |
| 2011-2020 | -0.38 (-4.74 to 2.30) | 0.744251 |
| **Hispanic or Latino** | | |
| 1999-2007 | 8.72 (6.37 to 18.22) | 0.005199 |
| 2007-2014 | 1.89 (0.48 to 8.66) | 0.016397 |
| 2014-2018 | -3.48 (-8.09 to -0.27) | 0.029594 |
| 2018-2020 | 26.65 (19.16 to 33.96) | < 0.000001 |
| **NH Asian or Pacific Islander** | | |
| 1999-2006 | 9.67 (6.07 to 17.11) | 0.0016 |
| 2006-2018 | -0.27 (-3.94 to 0.69) | 0.5003 |
| 2018-2020 | 12.55 (3.64 to 17.58) | < 0.000001 |
| **Nonmetropolitan areas** | | |
| 1999-2002 | 17.09 (12.62 to 27.19) | < 0.000001 |
| 2002-2008 | 8.57 (3.99 to 10.18) | 0.011198 |
| 2008-2018 | 1.14 (-1.44 to 1.89) | 0.147171 |
| 2018-2020 | 13.17 (6.39 to 16.94) | < 0.000001 |
| **Metropolitan area** | | |
| 1999-2005 | 10.37 (8.68 to 13.52) | < 0.000001 |
| 2005-2013 | 2.82 (1.99 to 3.87) | < 0.000001 |
| 2013-2018 | -1.70 (-5.02 to -0.51) | 0.005199 |
| 2018-2020 | 13.18 (8.98 to 16.82) | < 0.000001 |
| **Northeast region** | | |
| 1999-2003 | 13.78 (9.66 to 23.53) | < 0.000001 |
| 2003-2013 | 4.71 (3.82 to 5.58) | 0.0004 |
| 2013-2018 | -2.19 (-5.19 to -0.76) | 0.004399 |
| 2018-2020 | 15.65 (8.54 to 20.77) | < 0.000001 |
| **South region** | | |
| 1999-2005 | 11.15 (9.39 to 15.76) | < 0.000001 |
| 2005-2010 | 3.78 (2.05 to 7.39) | 0.0008 |
| 2010-2018 | 0.59 (-1.48 to 1.21) | 0.261148 |
| 2018-2020 | 16.77 (13.07 to 19.88) | < 0.000001 |
| **Midwest region** | | |
| 1999-2007 | 9.70 (8.01 to 12.10) | < 0.000001 |
| 2007-2020 | 0.75 (0.12 to 1.33) | 0.022795 |
| **West region** | | |
| 1999-2005 | 9.40 (7.57 to 12.99) | 0.0004 |
| 2005-2013 | 2.44 (1.43 to 3.63) | 0.0012 |
| 2013-2018 | -2.87 (-6.53 to -1.45) | 0.002799 |
| 2018-2020 | 8.68 (3.30 to 12.89) | 0.002799 |
| APC = annual percent change; NH = non-Hispanic; * Indicates that the annual percentage change (APC) is significantly different from zero at α = 0.05. AAMR = age-adjusted mortality rate. | | |

**Supplemental Table 4: Overall and Sex‐Stratified Coronary Artery Disease Related Age-Adjusted Mortality Rates per 100,000 in Adults with Hyperlipidemia in the United States, 1999 to 2020.**

| **Age-Adjusted Rate (95% CI)** | | | |
| --- | --- | --- | --- |
| **Year** | **Men** | **Women** | **Overall** |
| **1999** | 5.5 (5.3 - 5.6) | 2.9 (2.8 - 3.0) | 4.1 (4.0 - 4.1) |
| **2000** | 6.3 (6.1 - 6.4) | 3.1 (3.0 - 3.2) | 4.5 (4.4 - 4.6) |
| **2001** | 7.3 (7.1 - 7.5) | 3.6 (3.5 - 3.7) | 5.2 (5.1 - 5.3) |
| **2002** | 8.5 (8.3 - 8.7) | 4.2 (4.0 - 4.3) | 6.0 (5.9 - 6.1) |
| **2003** | 9.2 (9.0 - 9.4) | 4.4 (4.2 - 4.5) | 6.5 (6.3 - 6.6) |
| **2004** | 9.7 (9.5 - 10.0) | 4.6 (4.5 - 4.7) | 6.8 (6.7 - 6.9) |
| **2005** | 10.9 (10.7 - 11.2) | 5.3 (5.2 - 5.5) | 7.7 (7.6 - 7.9) |
| **2006** | 11.4 (11.2 - 11.7) | 5.5 (5.3 - 5.6) | 8.0 (7.9 - 8.1) |
| **2007** | 12.1 (11.9 - 12.3) | 5.8 (5.7 - 5.9) | 8.5 (8.4 - 8.6) |
| **2008** | 12.7 (12.4 - 12.9) | 5.9 (5.8 - 6.1) | 8.8 (8.7 - 8.9) |
| **2009** | 12.6 (12.3 - 12.8) | 6.0 (5.8 - 6.1) | 8.8 (8.6 - 8.9) |
| **2010** | 13.4 (13.1 - 13.6) | 6.2 (6.0 - 6.3) | 9.2 (9.1 - 9.3) |
| **2011** | 13.8 (13.6 - 14.1) | 6.2 (6.0 - 6.3) | 9.4 (9.3 - 9.5) |
| **2012** | 13.7 (13.4 - 13.9) | 6.3 (6.2 - 6.4) | 9.4 (9.3 - 9.6) |
| **2013** | 14.2 (13.9 - 14.4) | 6.4 (6.3 - 6.5) | 9.7 (9.6 - 9.8) |
| **2014** | 14.0 (13.8 - 14.3) | 6.3 (6.2 - 6.5) | 9.6 (9.5 - 9.8) |
| **2015** | 14.0 (13.7 - 14.2) | 6.3 (6.2 - 6.4) | 9.6 (9.4 - 9.7) |
| **2016** | 14.0 (13.8 - 14.2) | 6.3 (6.2 - 6.5) | 9.7 (9.5 - 9.8) |
| **2017** | 13.7 (13.5 - 14.0) | 6.1 (6.0 - 6.2) | 9.4 (9.2 - 9.5) |
| **2018** | 13.9 (13.7 - 14.1) | 6.0 (5.9 - 6.1) | 9.4 (9.3 - 9.5) |
| **2019** | 14.4 (14.2 - 14.6) | 6.3 (6.2 - 6.4) | 9.8 (9.7 - 9.9) |
| **2020** | 17.7 (17.4 - 17.9) | 7.9 (7.7 - 8.0) | 12.1 (12.0 - 12.3) |
| **Total** | **12.4 (12.4 - 12.5)** | **5.6 (5.6 - 5.6)** | **8.5 (8.5 - 8.5)** |

**Supplemental Table 5: Race‐Stratified Coronary Artery Disease-Related Age-Adjusted Mortality Rates per 100,000 in Adults with Hyperlipidemia in the United States, 1999 to 2020.**

| **Age-Adjusted Rate (95% CI)** | | | | | |
| --- | --- | --- | --- | --- | --- |
| **Year** | **NH White** | **NH Black or African American** | **NH Asian or Pacific Islander** | **NH American Indian or Alaska Native** | **Hispanic or Latino** |
| **1999** | 4.2 (4.1 - 4.3) | 3.4 (3.1 - 3.7) | 2.9 (2.4 - 3.4) | 2.6 (1.6 - 4.0) | 3.1 (2.7 - 3.4) |
| **2000** | 4.7 (4.6 - 4.8) | 3.7 (3.4 - 4.0) | 3.4 (2.8 - 3.9) | 4.8 (3.4 - 6.6) | 3.3 (2.9 - 3.6) |
| **2001** | 5.5 (5.4 - 5.6) | 4.0 (3.7 - 4.3) | 4.0 (3.4 - 4.6) | 3.9 (2.6 - 5.6) | 3.8 (3.4 - 4.3) |
| **2002** | 6.3 (6.2 - 6.4) | 4.8 (4.5 - 5.2) | 4.6 (3.9 - 5.2) | 4.4 (3.1 - 6.2) | 4.5 (4.1 - 4.9) |
| **2003** | 6.8 (6.6 - 6.9) | 5.3 (4.9 - 5.6) | 4.9 (4.3 - 5.5) | 5.1 (3.7 - 6.9) | 4.5 (4.0 - 4.9) |
| **2004** | 7.2 (7.0 - 7.3) | 5.6 (5.2 - 5.9) | 4.6 (4.0 - 5.2) | 5.8 (4.3 - 7.9) | 4.6 (4.2 - 5.0) |
| **2005** | 8.1 (8.0 - 8.3) | 6.2 (5.8 - 6.6) | 5.5 (4.8 - 6.1) | 7.2 (5.5 - 9.3) | 5.5 (5.0 - 5.9) |
| **2006** | 8.4 (8.2 - 8.5) | 6.7 (6.3 - 7.1) | 6.1 (5.4 - 6.7) | 7.6 (5.8 - 9.8) | 5.7 (5.3 - 6.2) |
| **2007** | 8.9 (8.7 - 9.0) | 7.1 (6.7 - 7.5) | 5.6 (5.0 - 6.2) | 8.9 (7.0 - 11.2) | 6.3 (5.8 - 6.7) |
| **2008** | 9.2 (9.1 - 9.4) | 7.4 (7.0 - 7.8) | 6.0 (5.4 - 6.6) | 7.1 (5.5 - 9.1) | 6.2 (5.8 - 6.7) |
| **2009** | 9.2 (9.1 - 9.4) | 7.5 (7.0 - 7.9) | 6.2 (5.6 - 6.8) | 8.4 (6.6 - 10.5) | 6.4 (6.0 - 6.8) |
| **2010** | 9.7 (9.5 - 9.8) | 7.9 (7.5 - 8.3) | 6.0 (5.5 - 6.6) | 9.0 (7.2 - 11.2) | 7.0 (6.5 - 7.4) |
| **2011** | 9.9 (9.8 - 10.1) | 7.7 (7.3 - 8.1) | 6.0 (5.4 - 6.5) | 11.5 (9.4 - 13.6) | 6.8 (6.4 - 7.2) |
| **2012** | 10.0 (9.8 - 10.1) | 8.1 (7.7 - 8.5) | 6.2 (5.6 - 6.7) | 11.1 (9.1 - 13.1) | 6.4 (6.0 - 6.9) |
| **2013** | 10.3 (10.1 - 10.4) | 8.3 (7.9 - 8.7) | 6.7 (6.1 - 7.2) | 9.5 (7.7 - 11.4) | 7.2 (6.8 - 7.6) |
| **2014** | 10.2 (10.1 - 10.4) | 7.9 (7.5 - 8.3) | 6.1 (5.6 - 6.6) | 9.9 (8.1 - 11.7) | 7.2 (6.8 - 7.6) |
| **2015** | 10.2 (10.1 - 10.3) | 8.1 (7.7 - 8.5) | 6.0 (5.5 - 6.5) | 10.0 (8.3 - 11.8) | 6.6 (6.3 - 7.0) |
| **2016** | 10.2 (10.0 - 10.3) | 8.7 (8.3 - 9.0) | 6.3 (5.8 - 6.8) | 10.6 (8.8 - 12.4) | 6.8 (6.5 - 7.2) |
| **2017** | 10.0 (9.8 - 10.1) | 8.1 (7.7 - 8.5) | 6.0 (5.5 - 6.4) | 9.5 (7.9 - 11.1) | 6.8 (6.5 - 7.2) |
| **2018** | 10.0 (9.9 - 10.2) | 8.4 (8.0 - 8.8) | 5.7 (5.3 - 6.1) | 9.4 (7.8 - 10.9) | 6.3 (6.0 - 6.7) |
| **2019** | 10.4 (10.3 - 10.6) | 8.9 (8.5 - 9.3) | 5.9 (5.4 - 6.3) | 8.9 (7.4 - 10.5) | 7.1 (6.8 - 7.5) |
| **2020** | 12.7 (12.5 - 12.9) | 11.3 (10.9 - 11.8) | 7.8 (7.3 - 8.2) | 12.1 (10.4 - 13.9) | 10.2 (9.8 - 10.6) |
| **Total** | **8.9 (8.9 - 8.9)** | **7.3 (7.2 - 7.4)** | **5.9 (5.8 - 6.0)** | **8.6 (8.2 - 9.0)** | **6.5 (6.4 - 6.6)** |
| NH = non-Hispanic. | | | | | |

**Supplemental Table 6: Coronary Artery Disease Related Age-Adjusted Mortality Rates per 100,000, Stratified by States in Adults with Hyperlipidemia, in the United States, 1999 to 2020**

| **State** | **Age-Adjusted Rate (95% CI)** |
| --- | --- |
| Alabama | 3.8 (3.7 - 4.0) |
| Alaska | 6.0 (5.3 - 6.6) |
| Arizona | 6.9 (6.7 - 7.0) |
| Arkansas | 5.6 (5.4 - 5.8) |
| California | 9.9 (9.8 - 10.0) |
| Colorado | 6.9 (6.7 - 7.1) |
| Connecticut | 5.6 (5.4 - 5.8) |
| Delaware | 10.9 (10.3 - 11.4) |
| District of Columbia | 4.6 (4.1 - 5.0) |
| Florida | 6.9 (6.9 - 7.0) |
| Georgia | 4.2 (4.1 - 4.3) |
| Hawaii | 11.7 (11.2 - 12.1) |
| Idaho | 8.0 (7.6 - 8.3) |
| Illinois | 6.2 (6.1 - 6.3) |
| Indiana | 9.6 (9.4 - 9.8) |
| Iowa | 12.1 (11.8 - 12.4) |
| Kansas | 7.3 (7.1 - 7.6) |
| Kentucky | 8.1 (7.9 - 8.3) |
| Louisiana | 5.2 (5.0 - 5.4) |
| Maine | 10.1 (9.7 - 10.5) |
| Maryland | 10.0 (9.8 - 10.2) |
| Massachusetts | 4.7 (4.6 - 4.8) |
| Michigan | 7.2 (7.1 - 7.4) |
| Minnesota | 10.2 (10.0 - 10.4) |
| Mississippi | 4.4 (4.2 - 4.6) |
| Missouri | 7.7 (7.5 - 7.8) |
| Montana | 6.6 (6.3 - 7.0) |
| Nebraska | 12.2 (11.8 - 12.6) |
| Nevada | 3.9 (3.7 - 4.1) |
| New Hampshire | 10.2 (9.7 - 10.6) |
| New Jersey | 8.6 (8.5 - 8.8) |
| New Mexico | 6.8 (6.5 - 7.1) |
| New York | 6.3 (6.3 - 6.4) |
| North Carolina | 9.7 (9.5 - 9.9) |
| North Dakota | 13.6 (12.9 - 14.3) |
| Ohio | 15.8 (15.7 - 16.0) |
| Oklahoma | 9.6 (9.3 - 9.8) |
| Oregon | 11.8 (11.5 - 12.0) |
| Pennsylvania | 9.6 (9.5 - 9.8) |
| Rhode Island | 12.5 (12.0 - 13.0) |
| South Carolina | 8.8 (8.6 - 9.0) |
| South Dakota | 8.8 (8.3 - 9.3) |
| Tennessee | 9.9 (9.7 - 10.1) |
| Texas | 9.8 (9.6 - 9.9) |
| Utah | 5.4 (5.2 - 5.7) |
| Vermont | 16.0 (15.3 - 16.8) |
| Virginia | 7.0 (6.9 - 7.2) |
| Washington | 10.8 (10.6 - 11.0) |
| West Virginia | 15.2 (14.8 - 15.7) |
| Wisconsin | 8.4 (8.3 - 8.6) |
| Wyoming | 9.7 (9.0 - 10.4) |

**Supplemental Table 7: Coronary Artery Disease related Age-Adjusted Mortality Rates per 100,000, Stratified by Census Region in Adults with Hyperlipidemia, in the United States, 1999 to 2020**

|  | **Census Region: NorthEast** | **Census Region: Midwest** | **Census Region: South** | **Census Region: West** |
| --- | --- | --- | --- | --- |
| **Year** | **Age-Adjusted Rate (95% CI)** | **Age-Adjusted Rate (95% CI)** | **Age-Adjusted Rate (95% CI)** | **Age-Adjusted Rate (95% CI)** |
| **1999** | 3.6 (3.4 - 3.8) | 4.5 (4.3 - 4.7) | 3.5 (3.3 - 3.6) | 5.1 (4.8 - 5.3) |
| **2000** | 4.0 (3.8 - 4.2) | 5.2 (5.0 - 5.4) | 4.0 (3.8 - 4.1) | 5.3 (5.1 - 5.5) |
| **2001** | 4.5 (4.3 - 4.7) | 5.9 (5.7 - 6.1) | 4.7 (4.6 - 4.9) | 6.0 (5.8 - 6.3) |
| **2002** | 5.2 (5.0 - 5.5) | 6.8 (6.5 - 7.0) | 5.3 (5.1 - 5.5) | 7.2 (7.0 - 7.5) |
| **2003** | 5.8 (5.5 - 6.0) | 7.2 (7.0 - 7.5) | 5.8 (5.6 - 6.0) | 7.5 (7.2 - 7.7) |
| **2004** | 6.1 (5.8 - 6.3) | 7.9 (7.6 - 8.1) | 6.1 (5.9 - 6.3) | 7.6 (7.3 - 7.9) |
| **2005** | 6.7 (6.5 - 7.0) | 9.1 (8.8 - 9.4) | 6.9 (6.7 - 7.1) | 8.6 (8.4 - 8.9) |
| **2006** | 6.7 (6.5 - 7.0) | 9.3 (9.1 - 9.6) | 7.2 (7.0 - 7.4) | 9.0 (8.7 - 9.3) |
| **2007** | 7.3 (7.0 - 7.6) | 10.0 (9.7 - 10.3) | 7.7 (7.5 - 7.9) | 9.3 (9.0 - 9.6) |
| **2008** | 7.5 (7.3 - 7.8) | 10.7 (10.4 - 11.0) | 7.7 (7.5 - 7.9) | 9.6 (9.3 - 9.9) |
| **2009** | 7.6 (7.4 - 7.9) | 10.2 (10.0 - 10.5) | 8.1 (7.9 - 8.3) | 9.4 (9.1 - 9.7) |
| **2010** | 8.4 (8.1 - 8.7) | 10.4 (10.1 - 10.7) | 8.4 (8.2 - 8.6) | 10.0 (9.7 - 10.3) |
| **2011** | 8.5 (8.2 - 8.8) | 10.9 (10.7 - 11.2) | 8.3 (8.1 - 8.5) | 10.4 (10.1 - 10.7) |
| **2012** | 9.0 (8.7 - 9.3) | 10.6 (10.3 - 10.9) | 8.4 (8.2 - 8.6) | 10.3 (10.0 - 10.6) |
| **2013** | 9.3 (9.0 - 9.6) | 10.8 (10.6 - 11.1) | 8.8 (8.6 - 9.0) | 10.5 (10.2 - 10.8) |
| **2014** | 9.2 (9.0 - 9.5) | 11.2 (10.9 - 11.5) | 8.6 (8.4 - 8.8) | 10.1 (9.8 - 10.4) |
| **2015** | 8.9 (8.6 - 9.2) | 11.1 (10.8 - 11.4) | 8.7 (8.5 - 8.9) | 10.0 (9.8 - 10.3) |
| **2016** | 8.9 (8.6 - 9.2) | 11.0 (10.7 - 11.2) | 8.9 (8.7 - 9.1) | 10.1 (9.9 - 10.4) |
| **2017** | 8.4 (8.1 - 8.7) | 11.2 (10.9 - 11.5) | 8.7 (8.5 - 8.8) | 9.6 (9.3 - 9.8) |
| **2018** | 8.8 (8.5 - 9.0) | 10.8 (10.5 - 11.1) | 8.9 (8.7 - 9.1) | 9.2 (9.0 - 9.5) |
| **2019** | 9.1 (8.8 - 9.4) | 10.8 (10.5 - 11.0) | 9.8 (9.6 - 10.0) | 9.4 (9.1 - 9.6) |
| **2020** | 12.1 (11.8 - 12.4) | 13.1 (12.8 - 13.4) | 12.2 (11.9 - 12.4) | 11.1 (10.9 - 11.4) |
| **Total** | **7.7 (7.6 - 7.7)** | **9.7 (9.6 - 9.7)** | **7.8 (7.8 - 7.9)** | **9.1 (9.1 - 9.2)** |

**Supplemental Table 8: Coronary Artery Disease Related Age-Adjusted Mortality Rates per 100,000, Stratified by Urban-Rural Classification in Adults with Hyperlipidemia, in the United States, 1999 to 2020.**

| **Age-Adjusted Rate (95% CI)** | | |
| --- | --- | --- |
| **Year** | **Metropolitan** | **Nonmetropolitan** |
| **1999** | 4.1 (4.0 - 4.2) | 4.1 (3.8 - 4.3) |
| **2000** | 4.6 (4.4 - 4.7) | 4.4 (4.2 - 4.7) |
| **2001** | 5.3 (5.1 - 5.4) | 5.1 (4.8 - 5.3) |
| **2002** | 5.9 (5.8 - 6.1) | 6.3 (6.0 - 6.6) |
| **2003** | 6.3 (6.2 - 6.5) | 6.9 (6.6 - 7.2) |
| **2004** | 6.7 (6.5 - 6.8) | 7.3 (7.1 - 7.6) |
| **2005** | 7.5 (7.4 - 7.7) | 8.5 (8.1 - 8.8) |
| **2006** | 7.9 (7.7 - 8.0) | 8.5 (8.2 - 8.8) |
| **2007** | 8.2 (8.1 - 8.4) | 9.5 (9.2 - 9.8) |
| **2008** | 8.5 (8.4 - 8.7) | 9.9 (9.6 - 10.2) |
| **2009** | 8.5 (8.3 - 8.6) | 10.1 (9.8 - 10.5) |
| **2010** | 8.9 (8.8 - 9.1) | 10.5 (10.2 - 10.8) |
| **2011** | 9.1 (9.0 - 9.3) | 10.7 (10.3 - 11.0) |
| **2012** | 9.2 (9.0 - 9.3) | 10.7 (10.3 - 11.0) |
| **2013** | 9.5 (9.3 - 9.6) | 11.0 (10.6 - 11.3) |
| **2014** | 9.3 (9.2 - 9.4) | 11.3 (11.0 - 11.7) |
| **2015** | 9.2 (9.1 - 9.3) | 11.5 (11.1 - 11.8) |
| **2016** | 9.4 (9.2 - 9.5) | 11.1 (10.8 - 11.4) |
| **2017** | 9.0 (8.9 - 9.1) | 11.1 (10.8 - 11.5) |
| **2018** | 8.9 (8.8 - 9.1) | 11.6 (11.3 - 11.9) |
| **2019** | 9.3 (9.2 - 9.4) | 12.2 (11.9 - 12.6) |
| **2020** | 11.5 (11.4 - 11.7) | 15.0 (14.7 - 15.4) |
| **Total** | **8.3 (8.2 - 8.3)** | **9.6 (9.6 - 9.7)** |
